# Exploring the Role of Extracellular Vesicles in the Pathogenesis of Tuberculosis

**DOI:** 10.3390/genes15040434

**Published:** 2024-03-29

**Authors:** Rakesh Arya, Hemlata Shakya, Reetika Chaurasia, Md Azizul Haque, Jong-Joo Kim

**Affiliations:** 1 Department of Biotechnology, Yeungnam University, Gyeongsan 38541, Republic of Korea; rakesharya101@yu.ac.kr (R.A.); azizul@ynu.ac.kr (M.A.H.); 2 Department of Biomedical Engineering, Shri G. S. Institute of Technology and Science, Indore 452003, Madhya Pradesh, India; hemlata.shakya19@gmail.com; 3 Department of Internal Medicine, Section of Infectious Diseases, Yale University School of Medicine, New Haven, CT 06519, USA

**Keywords:** *Mycobacterium tuberculosis*, VAMP5, diagnosis, biomarker, molecular target

## Abstract

Tuberculosis (TB) remains a significant global health concern, necessitating accurate diagnosis and treatment monitoring. Extracellular vesicles (EVs), including exosomes, play crucial roles in disease progression, with their associated genes serving as potential biomarkers and therapeutic targets. Leveraging publicly available RNA-Seq datasets of TB patients and healthy controls (HCs), to identify differentially expressed genes (DEGs) and their associated protein–protein interaction networks and immune cell profiles, the common EV-related DEGs were identified and validated in the GSE42830 and GSE40553 datasets. We have identified nine common EV-related DEGs (SERPINA1, TNFAIP6, MAPK14, STAT1, ITGA2B, VAMP5, CTSL, CEACAM1, and PLAUR) upregulated in TB patients. Immune cell infiltration analysis revealed significant differences between TB patients and HCs, highlighting increased proportions of various immune cells in TB patients. These DEGs are involved in crucial cellular processes and pathways related to exocytosis and immune response regulation. Notably, VAMP5 exhibited excellent diagnostic performance (AUC—0.993, sensitivity—93.8%, specificity—100%), with potential as a novel biomarker for TB. The EV-related genes can serve as novel potential biomarkers that can distinguish between TB and HCs. VAMP5, which functions in exosome biogenesis and showed significant upregulation in TB, can be targeted for therapeutic interventions and treatment outcomes.

## 1. Introduction

Despite several advancements in anti-TB therapy and vaccine immunization, TB remains a fatal disease that infects about one-fourth of the world’s population. It is a chronic inflammatory disease caused by *Mycobacterium tuberculosis* (*Mtb*). About 10.6 million people (95% uncertainty interval (UI): 9.9–11.4 million) developed TB in 2022. Worldwide, TB caused an estimated 1.30 million deaths (95% UI: 1.18–1.43 million). COVID-related disruptions are estimated to have resulted in almost half a million excess deaths from TB in the years 2020–2022 [1]. The traditional diagnostic methods for diagnosing pulmonary tuberculosis (PTB), such as microscopic examination of acid-fast bacilli and sputum cultures, are time-consuming, have low sensitivity (12–15%), and cannot be distinguished from other acid-fast bacilli [2]. Chest X-ray imaging can help diagnose pulmonary TB infection. However, it cannot detect latent TB infection (LTBI) [3]. Other molecular methods, such as GeneXpert MTB/RIF and DNA sequencing, require high-end instruments and expertise and can lead to false negative or false positive results [4]. Also, current methods of TB diagnostics are not able to predict reactivation disease progression from LTBI. Therefore, simple, specific, sensitive, non-sputum-based, rapid, and precise diagnostic methods for active TB need to be developed to improve diagnostic efficiency and reduce transmission rates [5]. In the last decade, biomarkers derived from blood, saliva, urine, etc., have been studied intensively for the development of novel diagnostic methods for PTB [6,7]. Blood-based biomarker diagnostics have the advantages of quick sample collection, quantification, and point-of-care (POC) tests and are used for predicting active TB infections and determining the progression of *Mtb* infections in individuals who are at risk of developing active TB [8,9,10,11,12]. However, identification of whole-blood RNA biosignature-based effective biomarkers is still lacking in the current scenario.

**Figure 1:**
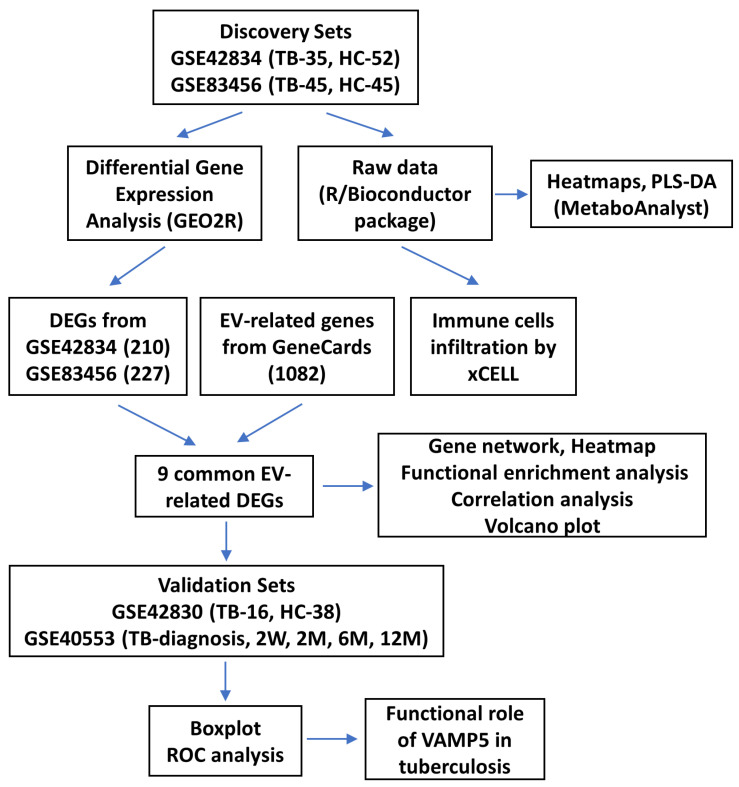
The schematic flowchart of the study. TB, tuberculosis; HC, healthy control; PLS-DA, partial least squares–discriminant analysis.

Extracellular vesicles (EVs) are membrane-bound vesicles that are secreted by almost all cell types and contain cargo of proteins, nucleic acids, etc., from the parent cell [13]. Exosomes are extracellular vesicles which vary from 50 to 200 nm in size and are responsible for delivering cargo to nearby cells through cell–cell communication [13]. The cargo contained in the EVs represents not only the original contents but also the perturbations during stress or disease conditions such as *Mtb* infection [14]. The EVs’ ability to encapsulate hydrophobic compounds, lipids, proteins, and glycolipids allows for distant interactions with the host that may play a vital role in the pathogenesis of TB [15,16]. In addition, they also function in various processes such as inhibition of antigen presentation, vaccination, TB biomarkers, recruitment of immune cells, and activation of macrophages [17]. Therefore, EVs and exosomes offer excellent biomarkers for TB prediction and prognosis, efficacy evaluation, etc. [18]. However, the functional role of EVs in TB disease and their relationship are still unclear. Therefore, to investigate the pathophysiology of TB infection using EVs, we have reanalyzed the freely available data of active TB and healthy controls from the Gene Expression Omnibus (GEO) database. We have identified EV-related DEG biosignatures that were deregulated in TB patients as compared to HCs. These gene expression data showed that innate immune cells were infiltrated in TB patients, which might be considered therapeutic targets of TB. We have also found that the EV-related gene VAMP5 is significantly upregulated in TB patients as compared to healthy controls and can serve a as potential biomarker for TB disease. The detailed study of EV-related genes in TB will provide an insight into the molecular mechanisms, can be potential biomarkers and targets for treatment, and can aid in the development of successful treatment outcomes. The workflow of the protocol used in the present study is described in Figure 1.

## 2. Materials and Methods

### 2.1. Datasets Collection

The RNA-seq data of human blood samples infected with TB from published microarray-based studies of TB versus healthy controls were downloaded from the Gene Expression Omnibus (GEO) database (https://www.ncbi.nlm.nih.gov/geo/, accessed on 19 October 2023) [19]. The GEOquery function of the R/Bioconductor package (ver. 4.3.0) was used to download the gene expression data from the datasets GSE42834, which consists of 35 pulmonary TB (PTB) and 52 HC samples [20], and GSE83456, which consists of 45 PTB and 45 HC samples [10], which were used as discovery sets. The other datasets, GSE42830, consisting of 16 PTB and 38 HC samples [20], and GSE40553, consisting of 131 samples of South African patients at different time points of diagnosis (2 weeks and 2, 6, and 12 months) after treatment [21], were used as validation sets. All four datasets are based on the same GPL10558 platform; Illumina HumanHT-12 V4.0 expression bead chip. All the datasets were downloaded from a public database, so informed consent and ethical approval were not needed. 

### 2.2. Differential Gene Expression (DEG) Identification

For raw data analysis, all four GEO datasets were downloaded as raw data matrix files containing microarray platform annotations from NCBI. MetaboAnalyst (ver. 5.0; https://www.metaboanalyst.ca/, accessed on 19 October 2023), an online tool, was used to analyze the raw data to generate heatmaps and PLS-DA (partial least squares–discriminant analysis) score plots [22]. We used quantile normalization and auto-scaling to perform the normalization and data scaling, respectively. For gene expression analysis, we used an NCBI-inbuilt tool, GEO2R (https://www.ncbi.nlm.nih.gov/geo/geo2r/, accessed on 19 October 2023), to identify the differentially expressed genes (DEGs) between the TB and control groups [19]. The *p*-value adjustment was performed using the Benjamini and Hochberg (false discovery rate) method. The data transformation was set to automatic mode, and limma precision weights (vooma) and data normalization were applied. The rows which did not have gene symbol information were not included in the analysis. For selecting the statistically significant DEGs from both the GSE42834 and GSE83456 datasets, we followed the filtering conditions of log_2_ (fold change) ≥±1 and *p*-value < 0.01.

### 2.3. Selection of Extracellular Vesicle (EV)-Related DEGs and Correlation Analysis

We downloaded the EV-related genes (n = 1082) from the GeneCards database (https://www.genecards.org/, accessed on 19 October 2023), which is a searchable, integrative database that provides comprehensive, user-friendly information on all annotated and predicted human genes [23]. All the EV-related genes involved in this study were selected with a relevance score ≥ 5. The DEGs from both discovery datasets, GSE42834 and GSE83456, and EV-related genes were used to find the common EV-related DEGs using a Venn diagram. To find the correlation among the 9 EV-related DEGs with a *p*-value < 0.05, we used SRplot, a freely available online visualization tool (https://bioinformatics.com.cn/, accessed on 19 October 2023) [24]. The expression levels of 9 EV-related DEGs were determined by heatmaps using MetaboAnalyst.

### 2.4. Assessment of Immune Cell Infiltration

xCell is an online tool that uses gene expression data to analyze 64 immune and stromal cell types. It uses gene signatures and reduces associations between cell types, outperforming previous methods and accurately depicting tissue expression profiles [25]. To calculate the difference in the proportion of different infiltrating immune cells in the immune microenvironment between the TB patients and HC samples, the xCell algorithm was used and the results were presented as a boxplot with a *p*-value < 0.05 (https://www.comphealth.ucsf.edu/app/xcell, accessed on 19 October 2023).

### 2.5. Functional Enrichment, Protein–Protein Interaction (PPI) Analysis, and Volcano Plot

To understand the functional role of all 9 EV-related DEGs, which were significantly enriched in certain pathways, we performed Gene Ontology (GO) of biological processes, molecular functions, cellular components, and pathway (KEGG) analysis using ShinyGO (ver. 0.77, http://bioinformatics.sdstate.edu/go/, accessed on 19 October 2023) [26,27]. To explore the mechanism of disease occurrence and development using functional interaction between genes/proteins, the PPI network of 9 EV-related DEGs was constructed using the online database Search Tool for the Retrieval of Interacting Genes (STRING, ver. 12; https://www.string-db.org/, accessed on 19 October 2023) [28]. The 9 EV-related DEGs were also presented in a volcano plot with a cutoff of log_2_ (fold change) ≥±1 and a *p*-value < 0.01, constructed using the VolcaNoseR online tool (https://huygens.science.uva.nl/VolcaNoseR/, accessed on 19 October 2023) [29].

### 2.6. Validation of EV-Related DEGs and ROC Analysis

All 9 EV-related DEG expression levels were validated in two independent datasets, GSE42830 and GSE40553, and shown as boxplots with interquartile ranges. The changes in gene expression in the GSE40553 dataset at different time points were also shown as a heatmap using MetaboAnalyst. Each EV-related DEG was evaluated to be considered a potential candidate for the diagnostic biomarker of TB by Receiver Operating Characteristic (ROC) curves using SRplot. The cutoff value for Area Under Curve (AUC) was 0.8, and a *p*-value < 0.05 was considered statistically significant.

### 2.7. Selection and Functional Role of VAMP5 as a Diagnostic Biomarker of TB

We followed the criteria of showing a high correlation with other EV-related DEGs, highly significant expression with a *p*-value < 0.0001 in both validation sets, function in vesicle-mediated transport, and a high AUC to select the VAMP5 gene, which can be a potential diagnostic biomarker of TB. To elucidate the functional role of VAMP5 in TB, we performed network interaction analysis of VAMP5 using the STRING database with a cut-off score > 0.7 and pathway analysis.

## 3. Results

### 3.1. Identification of DEGs between TB and Healthy Control Groups

**Figure 2:**
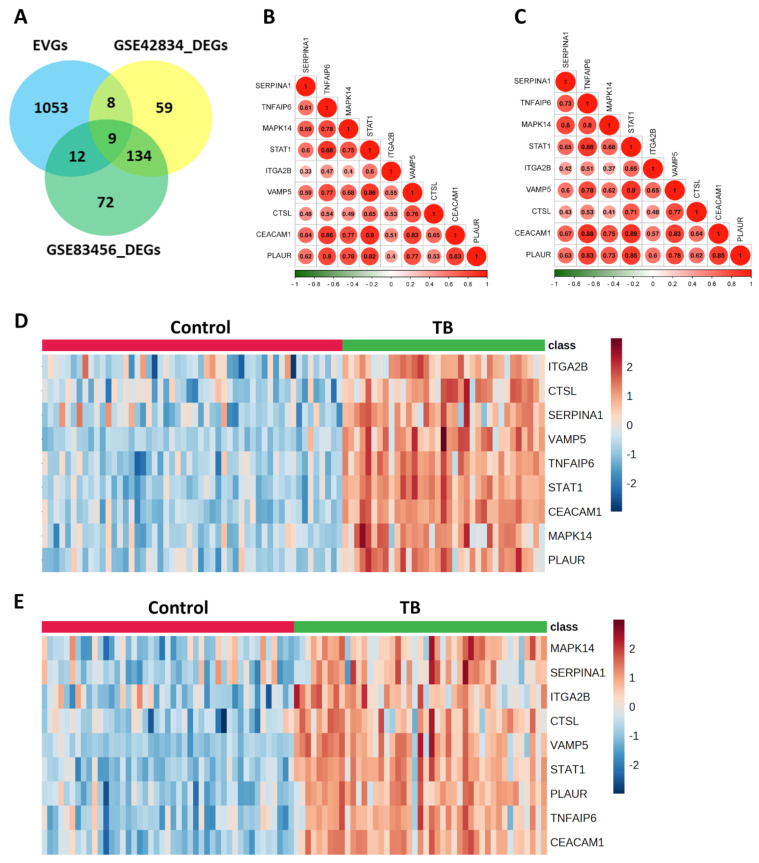
Screening, correlation, and heatmap analysis of EV-related gene signatures in GSE42834 and GSE83456 datasets. (**A**) The overlap of genes among EV-related genes from GeneCards (n = 1082) and DEGs of GSE42834 (n = 210) and GSE83456 (n = 227) datasets. (**B**,**C**) All 9 EV-related DEGs were positively correlated with each other in both datasets. (**D**,**E**) All 9 EV-related DEGs showed upregulation in TB patients as compared to HCs.

The analysis of the GSE42834 and GSE83456 datasets showed 31,266 features individually. In GSE42834, there were 52 healthy control samples and 35 samples from patients with TB, while GSE83456 had 45 healthy control samples and 45 TB patient samples. Both datasets showed distinct separation between the HC and TB sample groups, as indicated by the heatmaps and PLS-DA plots (Figure S1). Using GEO2R analysis, we screened 210 differentially expressed genes (DEGs) in GSE42834, among which 152 genes were upregulated and 58 genes were downregulated (Figure 2A). In addition, 227 DEGs were screened from GSE83456, among which 182 genes were upregulated and 45 genes were downregulated (Figure 2A).

### 3.2. Identification of EV-related DEG Signatures

We identified nine EV-related DEGs (SERPINA1, TNFAIP6, MAPK14, STAT1, ITGA2B, VAMP5, CTSL, CEACAM1, PLAUR) via the intersection of EV-related genes (n = 1082), GSE42834 DEGs (n = 210), and GSE83456 DEGs (n = 227) (Figure 2A). The nine EV-related DEGs were found to be highly correlated with each other (Figure 2B,C). Also, all nine EV-related DEGs were significantly upregulated in TB patient samples from both discovery sets (Figure 2D,E).

### 3.3. Alterations in Immune Cell Features between the TB and HC Groups

**Figure 3:**
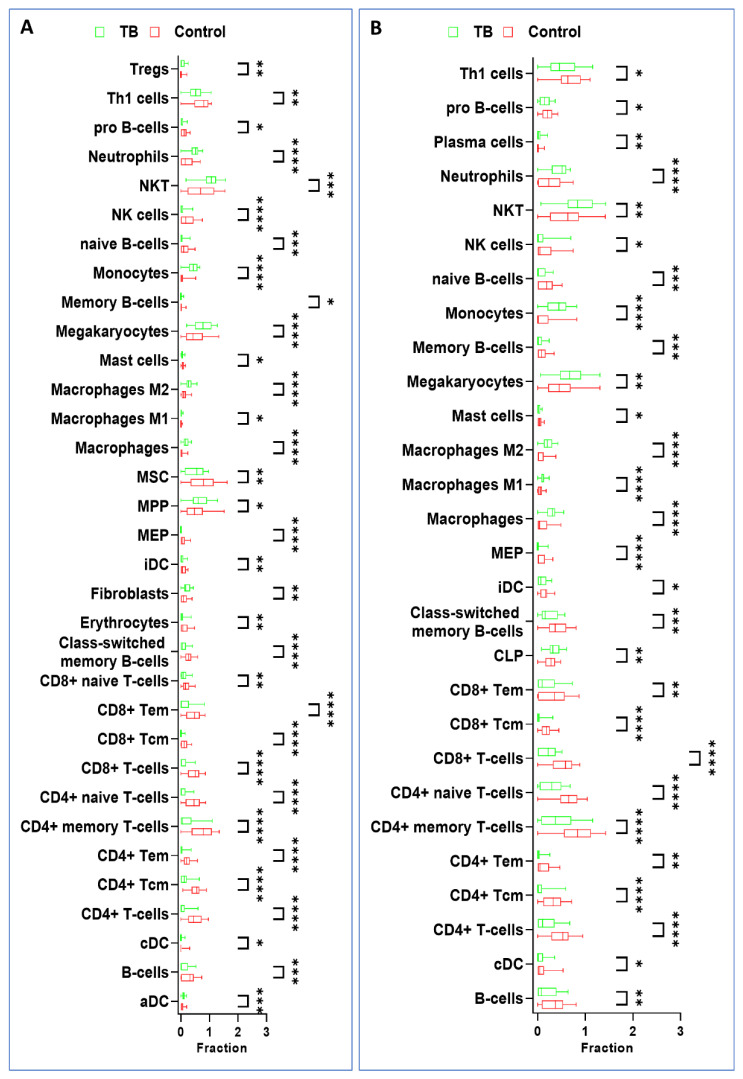
Immune cell infiltration in TB patients from GSE42834 and GSE83456 datasets. (**A**,**B**) The immune cell profiling of two datasets demonstrated that macrophages (M1, M2), monocytes, NKTs, neutrophils, Tregs cells, and plasma cells had a higher proportion in TB samples. * *p* < 0.05; ** *p* < 0.01; *** *p* < 0.001; **** *p* < 0.0001.

The xCell analysis between the TB and HC groups of the two discovery datasets highlighted significant differences in immune status. In the GSE42834 dataset, the TB patients had significantly higher numbers of aDCs, fibroblasts, multipotent progenitors (MPPs), macrophages, macrophages M1/M2, megakaryocytes, monocytes, NKTs, neutrophils, and Tregs cells compared to HCs. However, TB patients showed a significantly lower number of B-cells, cDCs, CD4+ T-cells, CD4+ Tcm, CD4+ Tem, CD4+ memory T-cells, CD4+ naive T-cells, CD8+ T-cells, CD8+ Tcm, CD8+ Tem, CD8+ naive T-cells, class-switched memory B-cells, erythrocytes, iDCs, megakaryocyte–erythroid progenitors (MEPs), mesenchymal stem cells (MSCs), mast cells, memory B-cells, naive B-cells, NK cells, pro B-cells, and Th1 cells compared to HCs. Also, in the GSE83456 dataset, the TB patients had significantly higher numbers of common lymphoid progenitors (CLPs), macrophages, macrophages M1/M2, megakaryocytes, monocytes, NKTs, neutrophils, and plasma cells as compared to HCs. However, TB patients showed significantly lower numbers of B-cells, cDCs, CD4+ T-cells, CD4+ Tcm, CD4+ Tem, CD4+ memory T-cells, CD4+ naive T-cells, CD8+ T-cells, CD8+ Tcm, CD8+ Tem, class-switched memory B-cells, iDCs, megakaryocyte–erythroid progenitor (MEPs), mast cells, memory B-cells, naive B-cells, NK cells, pro B-cells, and Th1 cells as compared to HCs (Figure 3). The results demonstrated that diverse types of immune cells, specifically innate immune cells, were uniquely infiltrated in TB patients, which might be considered as therapeutic targets of TB.

### 3.4. Functional Enrichment, PPI Network Analysis, and Volcano Plot

**Figure 4:**
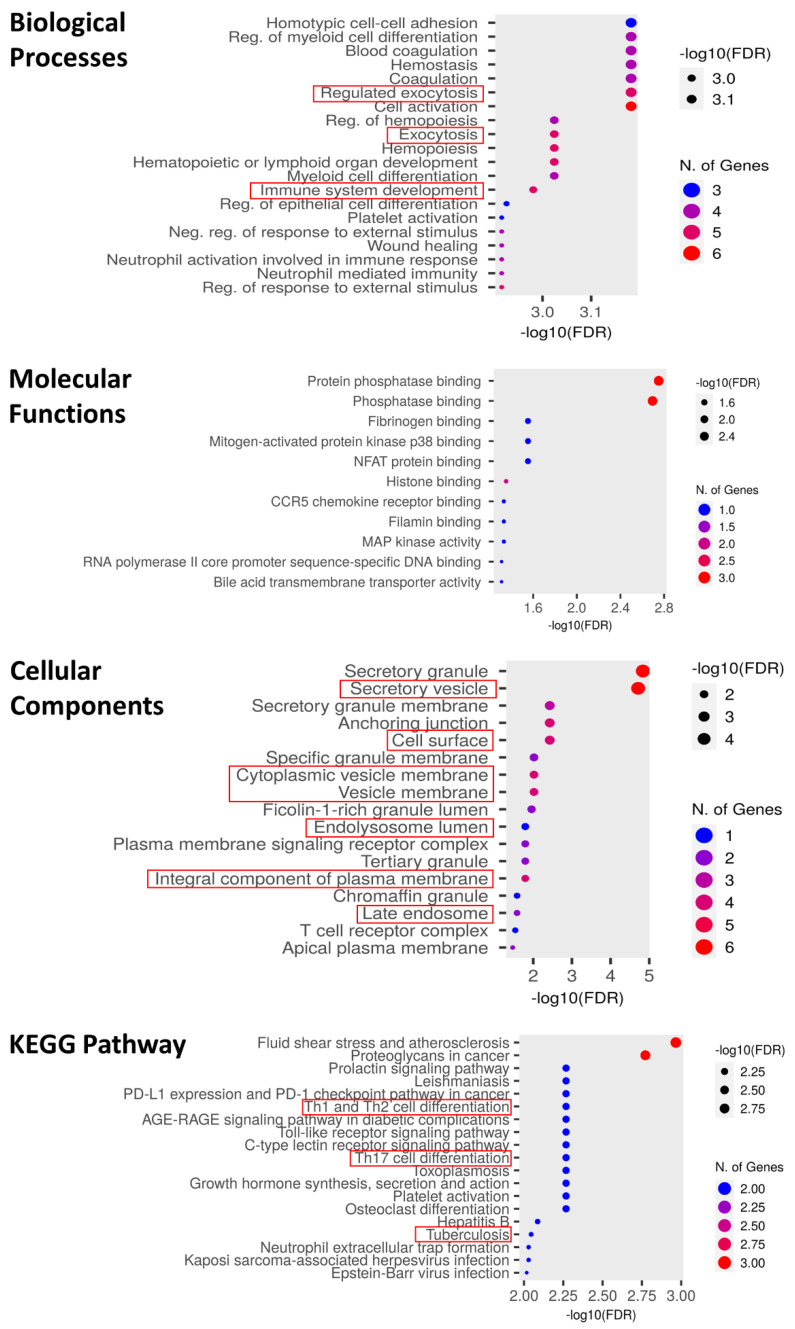
Functional enrichment analysis of EV-related DEGs. Enrichment of GO BP, GO MF, GO CC, and KEGG pathway analysis mainly associated with exocytosis, immune system development, late endosome, and Th1, Th2, and Th17 cell differentiation.

ShinyGO was employed to conduct GO and KEGG pathway analysis. The results showed enrichment of DEGs associated with different biological processes, such as regulated exocytosis, immune system development, and cellular components such as secretory vesicles, cytoplasmic vesicle membrane, late endosome, etc. (Figure 4). Also, the KEGG pathway indicated that these genes are involved in Th1, Th2, and Th17 cell differentiation, tuberculosis, etc. (Figure 4). The GO and KEGG pathway analysis indicated enrichment of the immune response, which played an important role in TB occurrence, progression, and regulation (Figure 4). The PPI (protein–protein interaction) network analysis was conducted on nine EV-related DEGs using STRING and showed that all the genes significantly interact with each other (Figure S2). The volcano plot showed that all nine genes showed significant upregulation in TB patients as compared to HC samples in both discovery datasets (Figure S2). The UniProt database was used to extract information about the functions and cellular components of these nine genes as shown in Table 1.

### 3.5. Validation and Detection of EV-Related DEG Expression during TB Treatment

**Figure 5:**
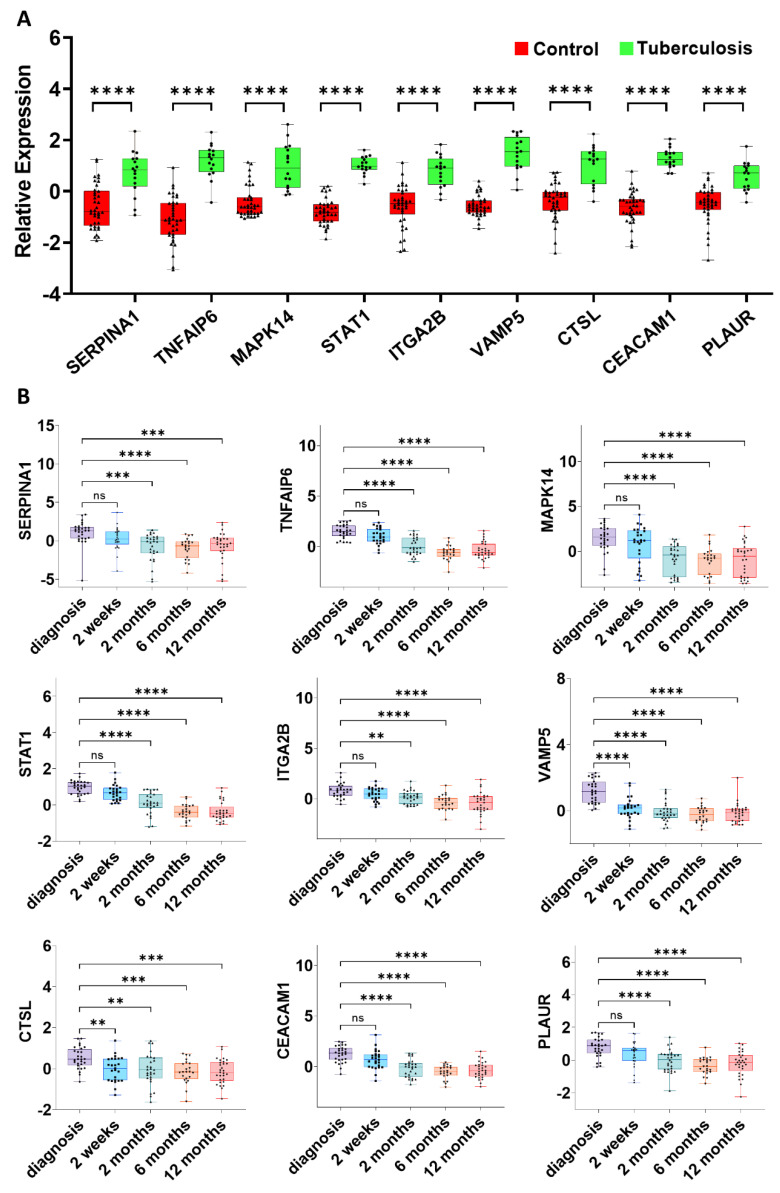
Validation of 9 EV-related DEGs in GSE42830 and GSE40553 datasets. (**A**) All genes showed significant upregulation in the expression levels between TB and HC groups. (**B**) All genes showed significant downregulation after treatment of 2 weeks and 2, 6, and 12 months. ** *p* < 0.01; *** *p* < 0.001; **** *p* < 0.0001; ns, nonsignificant.

The validation of gene expression in the GSE42830 dataset revealed significant differences in the expression levels of all nine EV-related DEGs between TB and healthy control groups (Figure 5A). To explore how the expression level changes during the TB treatment regimen, we examined another validation dataset, GSE40553, which included a total of 131 samples from 29 TB patients at five different time points: diagnosis, treatment for 2 weeks, and treatment for 2, 6, and 12 months. Heatmap analysis illustrated that the expression levels of all of the genes showed significant downregulation during TB treatment (Figure S3). Among these nine genes, VAMP5 showed a significant decrease in expression level during the TB treatment (Figure 5B). The expression level of VAMP5 significantly increased during TB infection and showed a significant decrease during treatment for 2 weeks and 2, 6, and 12 months. This suggests that VAMP5 could potentially serve as a valuable drug target for TB diagnosis.

### 3.6. Diagnostic Performance and Functional Role of VAMP5 in TB

The VAMP5 gene followed all four criteria, showing a high correlation with the other eight genes, highly significant expression in both validation sets, function in vesicle-mediated transport, and a high AUC of 0.986, 0.962, and 0.993 in the GSE42834, GSE83456, and GSE42830 datasets, respectively (Figure S4). The VAMP5 gene can be a potential candidate biomarker that can distinguish between TB patients and HCs, as its diagnostic performance can reach up to a sensitivity of 93.8% and a specificity of 100% in the GSE42830 dataset. The VAMP5 gene showed significant interaction with 10 interacting partner genes (interaction score > 0.7), GOSR1, NAPA, SNAP23, SNAP29, STX4, STX5, STX8, STX16, VAMP8, and YKT6, which are all part of the SNARE (Soluble N-ethylmaleimide-sensitive factor-activating protein receptor) family of genes that mediate the fusion and release of vesicles such as exosomes (Figure S5).

## 4. Discussion

With a higher rate of morbidity and death than other diseases, TB remains the deadliest disease in the world [1]. Treating TB is a challenging task that requires multiple pharmacological treatments over several months to achieve a long-lasting recovery [40]. Long-term treatment for TB is complex and multifactorial, with antibiotic resistance being a major challenge. The COVID-19 pandemic in 2020 has also exacerbated the diagnosis, treatment, and global TB burden [41,42]. There is, however, a lack of understanding of the molecular mechanism underlying the pathogenesis of TB. Therefore, novel biomarkers for quick and consistent TB diagnosis and monitoring are required to develop targeted treatment strategies utilizing these mechanisms.

EVs are crucial for biological processes, and their dysfunction is linked to various diseases. They carry molecules like DNA, mRNA, proteins, and lipids, which have already been used as potential biomarkers for *Mtb* infection [43]. However, their relevance to TB remains unclear. There have been many developments in the field of EV research for developing biomarkers of clinical importance using easily accessible biofluids such as blood and urine and harnessing their potential in the diagnosis and treatment of diseases [16,44]. To our knowledge, this is the first study to analyze the functions of EV-related DEGs in TB. We used the blood microarray datasets GSE42834 and GSE83456 as discovery sets and GSE42830 and GSE40553 as validation sets from the GEO database to identify EV-related candidate biomarkers in TB. The heatmaps and PLS-DA plots showed that the HC and TB groups were significantly distinct from each other. We identified a total of 210 DEGs (Up—152, Down—58) and 227 DEGs (Up—182, Down—45) between HC and TB patients in the GSE42834 and GSE83456 discovery datasets, respectively. To further confirm the relationship between nine EV-related DEGs (SERPINA1, TNFAIP6, MAPK14, STAT1, ITGA2B, VAMP5, CTSL, CEACAM1, and PLAUR) and TB, we determined the correlation, which showed that these genes interacted synergistically and were significantly upregulated in TB patients.

The immune infiltration analysis demonstrated that macrophages (M1, M2), monocytes, NKTs, neutrophils, Tregs cells, and plasma cells had higher numbers in TB samples in both datasets, which corroborated previous studies [45,46,47,48,49,50]. Human M1 macrophages express unique innate immune response genes after mycobacterial infection to defend against TB, while M2 macrophages have an inhibitory effect on immune responses, inhibiting antigen presentation and T-cell proliferation, exerting anti-inflammatory responses, attenuating cellular immunity against TB infection, and thereby promoting chronic TB infection [51,52]. During the onset of *Mtb* infection, monocytes are the primary innate immune cells that shield the host against intracellular infections [53]. The significance of immunity in the development of TB was demonstrated by each of these cells. The GO and KEGG enrichment analyses demonstrated that the nine EV-related DEGs were enriched in immune response development and Th1, Th2, and Th17 cell differentiation, which play an important role in the occurrence, progression, and regulation of TB. The PPI network showed significant interaction among all genes. All nine EV-related DEGs were validated in two independent datasets (GSE42830 and GSE40553), and the findings corroborated the discovery set results. Moreover, in GSE40553, the expression levels of all nine genes were down during TB treatment, which showed that TB treatment affects the levels of these genes and may be targeted for potential biomarkers of TB.

Out of these nine EV-related DEGs, VAMP5 was selected as it fulfilled the four criteria of showing high correlation with the other eight genes, highly significant expression in both validation sets, function in vesicle-mediated transport, and high AUC of 0.986, 0.962, and 0.993 in the GSE42834, GSE83456, and GSE42830 datasets, respectively. VAMP5, which functions as a key regulator of exosome release during exosome biogenesis, was found to be perturbed during TB infection and showed a significant decrease during TB treatment for 2 weeks and 2, 6, and 12 months. Also, it can serve as a potential diagnostic biomarker that can distinguish between HCs and TB patients, as its diagnostic performance can reach up to a sensitivity of 93.8% and a specificity of 100% in the GSE42830 dataset.

Matsui et al. showed that VAMP5 functions by regulating the release of exosomes from various cell types without affecting exosome biogenesis [36]. VAMP5 is most extensively co-localized with the exosome marker protein CD63 in polarized Madin–Darby canine kidney (MDCK) cells. Additionally, the isolated CD63-positive MVB sample contained endogenous VAMP5, suggesting that VAMP5 is probably an MVB-localized SNARE protein [36]. VAMP5 was demonstrated to be required for the translocation of glucose transporter 4 (GLUT4) to the plasma membrane [54].

**Figure 6:**
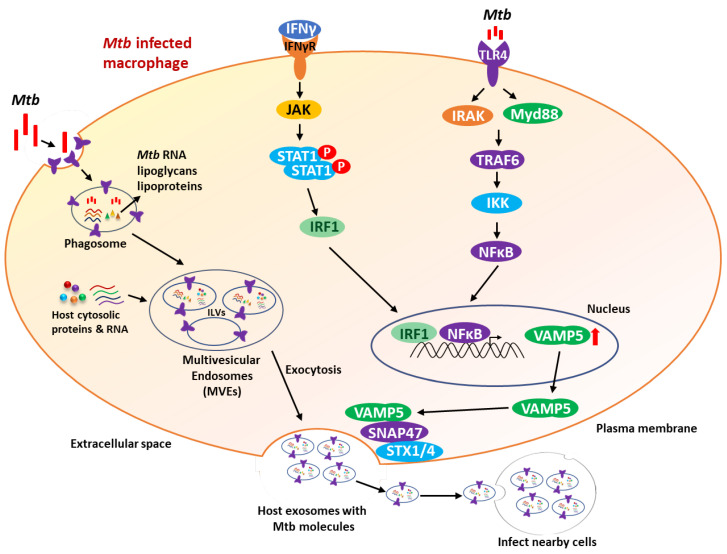
Role of VAMP5 in mediating the release of *Mtb*-specific molecules. The process starts with the infection of macrophages by *Mtb*, which enters into the intracellular space using various surface receptors. This also results in the induction of TLR4 and IFN-γ signaling pathways, which also triggers the upregulation of VAMP5 protein. The *Mtb* biomolecules such as RNA, lipoglycans, and lipoproteins are released into the phagosome and with the help of host cytosolic proteins and RNAs are transported to multivesicular endosomes (MVEs) and finally to inclusion bodies. The VAMP5 protein interacts with SNARE proteins such as synaptosome-associated protein (SNAP47) and syntaxin-1/4 (STX1/4), resulting in the excess release of exosomes containing *Mtb* molecules to infect nearby cells.

VAMP5 showed significant interaction with 10 interacting genes (GOSR1, NAPA, SNAP23, SNAP29, STX4, STX5, STX8, STX16, VAMP8, and YKT6) in the STRING database, and these are all part of the SNARE family of proteins that function in the biogenesis of exosomes. Out of these, SNAP47 was found to interact most strongly with VAMP5 and regulate exosome release together with VAMP5 [36], and was also described to be present at CD63-positive endosomes [55] and was involved in autophagy [56,57]. According to a recent study, VAMP5 was found to be present on the luminal side of MVBs and is released as a part of CD63-positive exosomes from Muller cells in the retina [58]. VAMP5 may be present on the MVB surface before intraluminal vesicle formation, leading to accidental incorporation into intraluminal vesicles (ILVs) with exosomal proteins like CD63, and together with SNAP47 and STX1/4, mediates MVB–plasma membrane fusion, resulting in its secretion as exosomes (Figure 6). Reduction in the levels of VAMP5 protein impaired exosome release from HeLa cells. VAMP5 is assumed to be translocated to the MVB surface via the endocytic pathway because VAMP5 is partially localized at the plasma membrane [36,59]. Another study indicated that alcohol increases the expression of synatxin-16, VAMP3, VAMP5, and VAMP7 in hepatocytes, and patients with alcohol-associated liver disease (ALD) also had higher levels of these SNAREs [60]. Another study revealed that the four-gene biosignature (BATF2, UBE2L6, VAMP5, and SERPING1) is a robust blood-based diagnostic for active TB across seven datasets containing more than 1200 clinical samples and showed that the sensitivity or specificity can reach up to 100% (mean AUC = 0.86, sensitivity = 86%, and specificity = 81%) [61]. A recent study showed that blood RNA sequencing of 10 pulmonary TB patients (4 drug-susceptible TB and 6 drug-resistant TB) identified 55 genes that were differentially expressed in MDR/RR-TB compared to drug-susceptible/mono-resistant TB. CD300LD, MYL9, VAMP5, CARD17, CLEC2B, GBP6, BATF2, ETV7, IFI27, and FCGR1CP showed upregulation in MDR/RR-TB in all comparisons, among which CLEC2B and CD300LD were not previously linked to TB [62].

*Mtb* releases biomolecules such as RNA, lipoglycans, lipoproteins, etc., within the phagosome, inserts them into host membranes, and transports them to multivesicular endosomes (MVEs), where they are integrated into inclusion vesicles (inclusion vesicles can also be generated within phagosomes by similar methods). Following MVE fusion with the plasma membrane, exosomes containing *Mtb* molecules are then released via exocytosis [63]. *Mtb* infection induces the IFN-γ and TLR4 pathways, which increases the level of VAMP5 protein. This VAMP5 protein, along with SNARE proteins such as synaptosome-associated protein (SNAP47) and syntaxin-1/4 (STX1/4), results in the excess release of exosomes containing *Mtb* molecules to infect other nearby cells during *Mtb* infection (Figure 6) [36].

The p38 signaling transduction pathway (a mitogen-activated protein kinase pathway), plays an important role in modulating the immune response of the host by coordinating the release of pro-inflammatory cytokines such as interleukin (IL-1β) or tumor necrosis factor (TNF) upon infection [64]. A recent study demonstrated that nine genes (CD274, CEACAM1, CR1, FCGR1A/B, IFITM1, IRAK3, LILRA6, MAPK14, and PDCD1LG2) showed 100% sensitivity and specificity that distinguished patients with active TB from those with other lung diseases (OPD). They also showed that seven genes (C1QB, C2, CCR2, CCRL2, LILRB4, MAPK14, and MSR1) were able to discriminate TB from LTBI with a sensitivity and specificity between 82 and 100% [38]. Another study demonstrated the critical role that the nuclear factor kappa B (NK-KB) pathway-regulated genes CXCL3, PTGS2, and TNFAIP6 played in the innate immunological stress response to TB [65]. The diagnostic accuracy of the seven-gene signature (Down—FCGBP and KLF12; Up—FCGR1B, ANKRD22, CARD17, IFITM3, and TNFAIP6) showed AUC values between 0.84 and 1 with a sensitivity of 80–100% and specificity of 80–95%, which can differentiate between active TB and LTBI [66].

There are various limitations to this study. First, the conclusions are purely based on bioinformatics analysis; neither experimental nor clinical validation is present. Second, additional validation is necessary, as the limited sample size might not fully capture the entire picture. Lastly, because the original sequencing data are not accessible and the data were obtained from a public repository, there is bias in the sample selection process. Also, extensive research is necessary to better validate the putative molecular mechanism underlying the pathogenesis of TB.

## 5. Conclusions

In conclusion, the EV-related gene biosignatures can play an important role in determining the clinical outcomes of TB patients. VAMP5 is significantly upregulated in TB patients upon infection, which indicates the key role of the VAMP5 protein in the TB immune process. Thus, the VAMP5 gene can be used as a potential diagnostic biomarker of TB disease and can predict the occurrence of TB. Also, in-depth analysis of VAMP5-interacting partner genes will give an insight into TB pathophysiology and can serve as potential biomarkers and treatment targets to promote the development of effective treatment outcomes.

## Figures and Tables

**Table 1:** The functions of the 9 EV-related DEGs.

Genes	Name	Functions (UniProt)	Cellular Component	References
SERPINA1	alpha-1-antitrypsin	Inhibitor of serine proteases. Its primary target is elastase, but it also has a moderate affinity for plasmin and thrombin. Irreversibly inhibits trypsin, chymotrypsin, and plasminogen activator. The aberrant form inhibits insulin-induced NO synthesis in platelets, decreases coagulation time, and has proteolytic activity against insulin and plasmin.	Extracellular exosome/region/space/matrix	[30]
TNFAIP6	Tumor necrosis factor-inducible gene 6 protein	Major regulator of extracellular matrix organization during tissue remodeling. Modulates the interaction of chemokines with extracellular matrix components and proteoglycans on endothelial cell surface, likely preventing chemokine gradient formation.	Extracellular region/space, cytoplasmic vesicle	[31]
MAPK14	Mitogen-activated protein kinase 14	Serine/threonine kinase which acts as an essential component of the MAP kinase signal transduction pathway. MAPK14 is one of the four p38 MAPKs which play an important role in the cascades of cellular responses evoked by extracellular stimuli such as pro-inflammatory cytokines or physical stress leading to direct activation of transcription factors.	Extracellular region, cytoplasmic vesicle	[32]
STAT1	Signal transducer and activator of transcription 1-alpha/beta	Signal transducer and transcription activator that mediates cellular responses to interferons (IFNs), cytokine KITLG/SCF, and other cytokines and other growth factors.		[33,34]
ITGA2B	Integrin alpha-IIb	Integrin alpha-IIb/beta-3 is a receptor for fibronectin, fibrinogen, plasminogen, prothrombin, thrombospondin, and vitronectin.	Extracellular exosome/space, cytoplasmic vesicle	[35]
VAMP5	Vesicle-associated membrane protein 5	May participate in trafficking events that are associated with myogenesis, such as myoblast fusion and/or GLUT4 trafficking.	Extracellular exosome/space, cytoplasmic vesicle	[36]
CTSL	Cathepsin L	Thiol protease is important for the overall degradation of proteins in lysosomes. Major elastin-degrading enzyme at neutral pH. Accumulates as a mature and active enzyme in the extracellular space of antigen-presenting cells (APCs) to regulate degradation of the extracellular matrix in the course of inflammation.	Extracellular exosome/region/space, collagen-containing extracellular matrix, cytoplasmic vesicle	[37]
CEACAM1	Carcinoembryonic antigen-related cell adhesion molecule 1	Cell adhesion protein that mediates homophilic cell adhesion in a calcium-independent manner, plays a role in immune response of T-cells, natural killer (NK), and neutrophils.	Extracellular exosome/space, cytoplasmic vesicle	[38]
PLAUR	Urokinase plasminogen activator surface receptor	Acts as a receptor for urokinase plasminogen activator and plays key roles in tissue remodeling and extracellular matrix degradation.	Extracellular region, cytoplasmic vesicle	[39]

## Data Availability

The datasets, including GSE42834, GSE83456, GSE42830, and GSE40553, are accessible from the NCBI Gene Expression Omnibus database (GEO, http://www.ncbi.nlm.nih.gov/geo, accessed on 19 October 2023).

## Supplementary Materials

The following supporting information can be downloaded at: https://www.mdpi.com/article/10.3390/genes15040434/s1, Figure S1. Heatmaps and PLS-DA analysis of two discovery datasets. Figure S2. Protein–protein interaction network and volcano plot analysis. Figure S3. Heatmap analysis showed significant downregulation of nine EV-related DEGs during TB treatment. Figure S4. ROC analysis of VAMP5 gene in GSE42834, GSE83456, and GSE42830 datasets. Figure S5. Protein–protein interaction network analysis of VAMP5 with 10 SNARE family genes.
