# Supplementary material for: Exploring the Role of Extracellular Vesicles in the Pathogenesis of Tuberculosis
Source: Genes (Basel). 2024 Mar 29;15(4):434. doi: 10.3390/genes15040434 (PMC11049626; doi:10.3390/genes15040434)
Supplement: Supplementary file 1 [file genes-15-00434-s001.zip › genes-2925023-supplementary.pdf]

Supplementary File

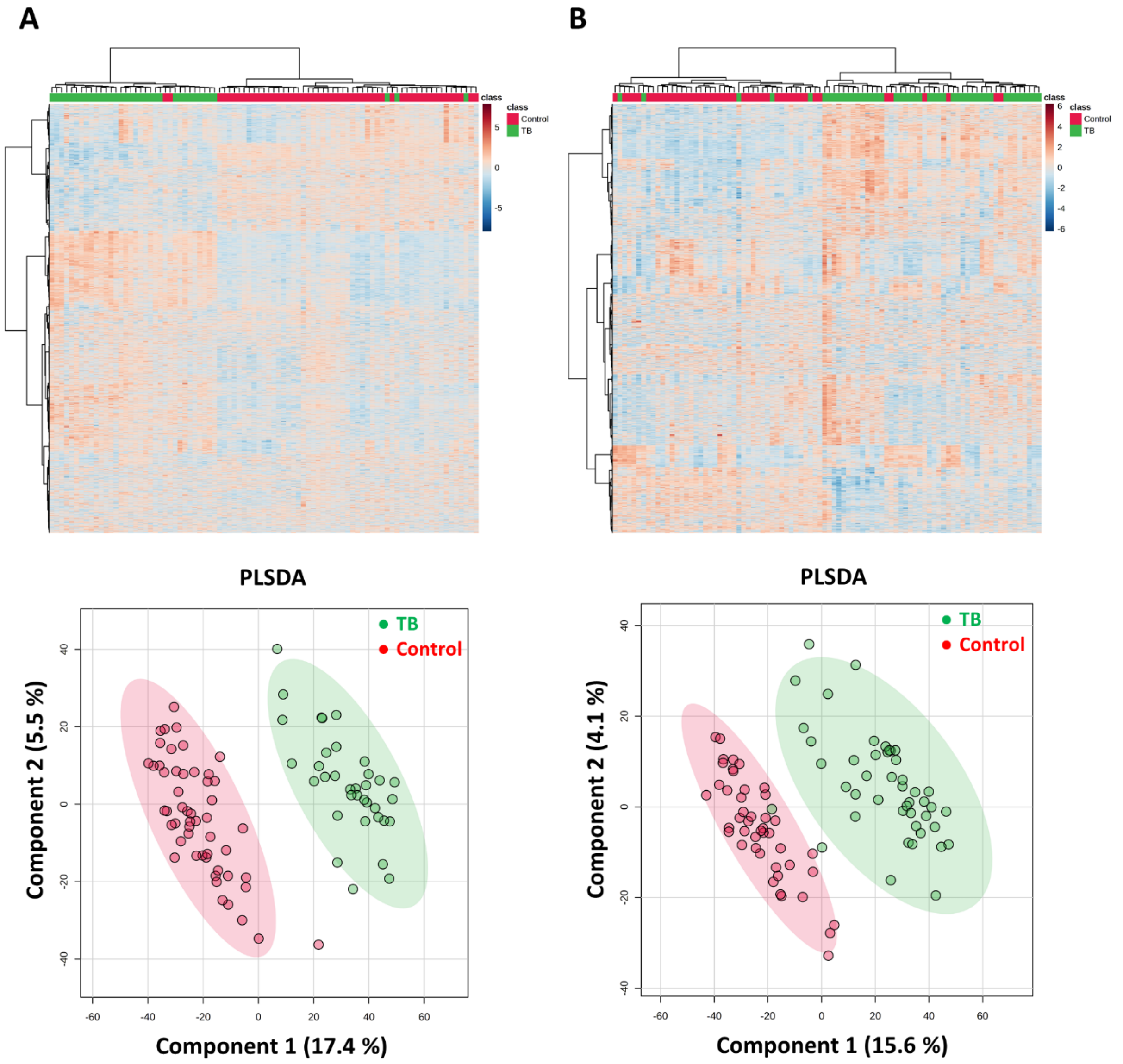

Figure S1. Heatmaps and PLS-DA analysis of two discovery datasets. (A) GSE42834, and (B) GSE83456, demonstrated good separation between TB and HC groups. The higher the percentage of components, the better is the separation between the two groups.

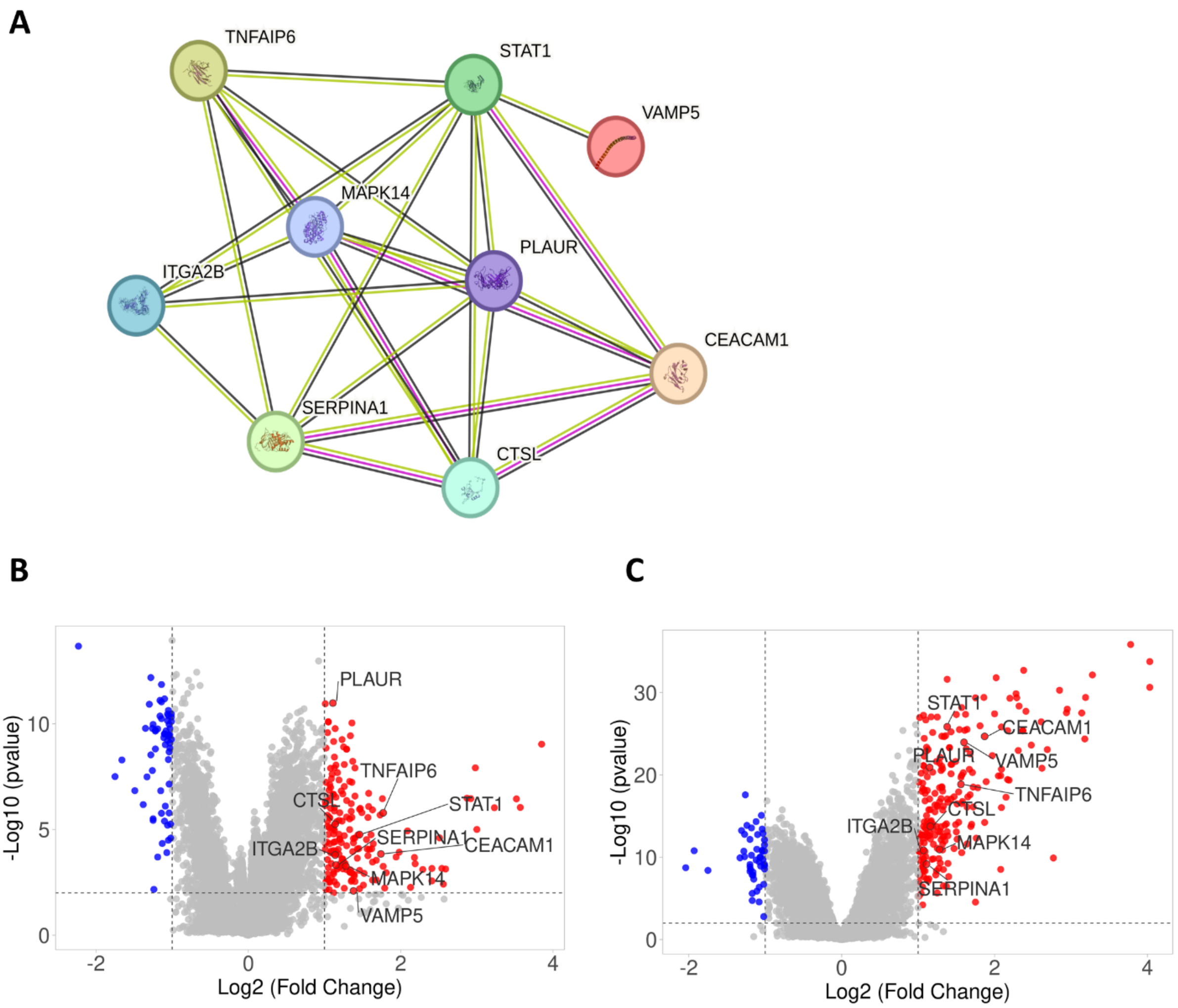

Figure S2. Protein-protein interaction network and volcano plot analysis. (A) The PPI network showed good interaction among all 9 EV-related DEGs. Volcano plot of (GSE42834), and (B) GSE83456, represented that all 9 EV-related DEGs were significantly upregulated in TB patients.

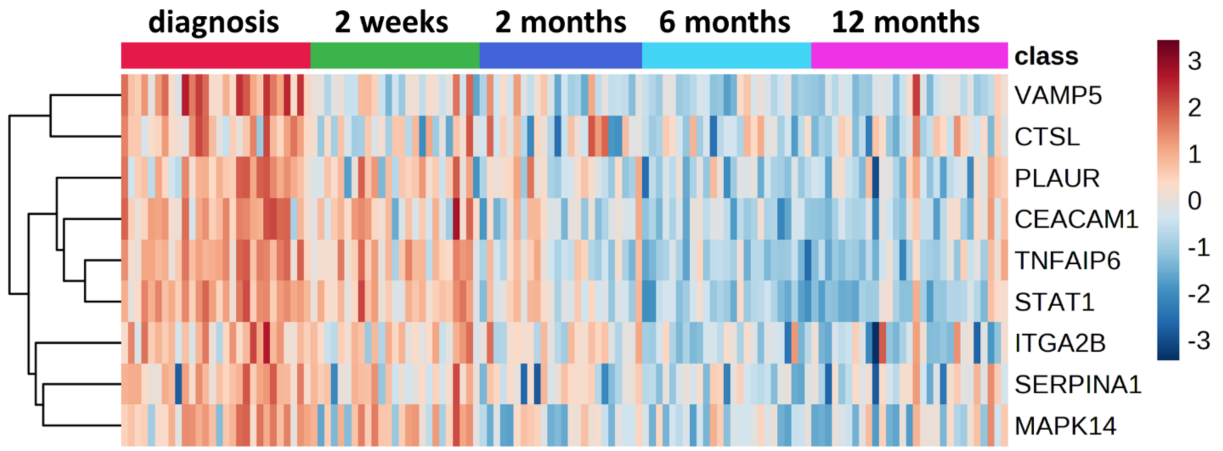

Figure S3. Heatmap analysis showed significant downregulation of 9 EV-related DEGs during TB treatment.

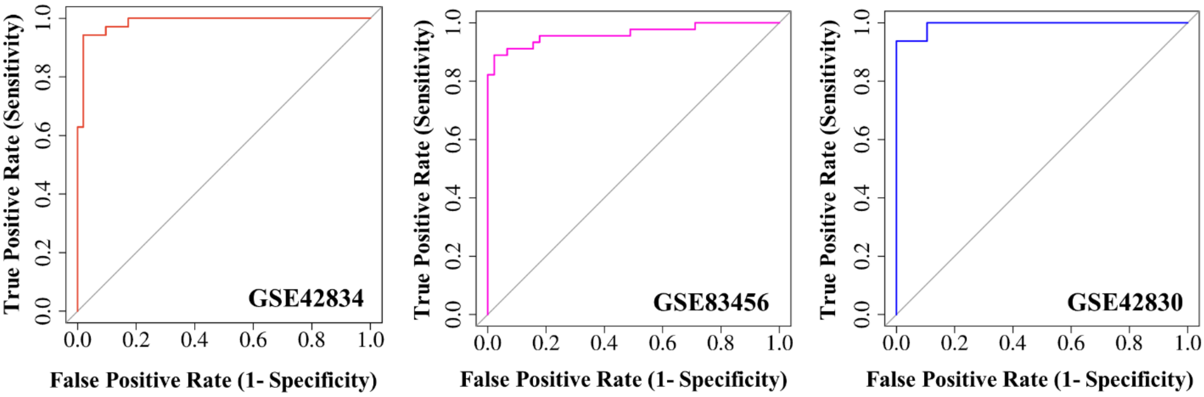

AUC (95% CI)	Sensitivity	Specificity
0.986 (0.968-1)	0.943	0.981

AUC (95% CI)	Sensitivity	Specificity
0.962 (0.923-1)	0.889	0.978

AUC (95% CI)	Sensitivity	Specificity
0.993 (0.965-1)	0.938	1

Figure S4. ROC analysis of VAMP5 gene in GSE42834, GSE83456, and GSE42830 datasets.

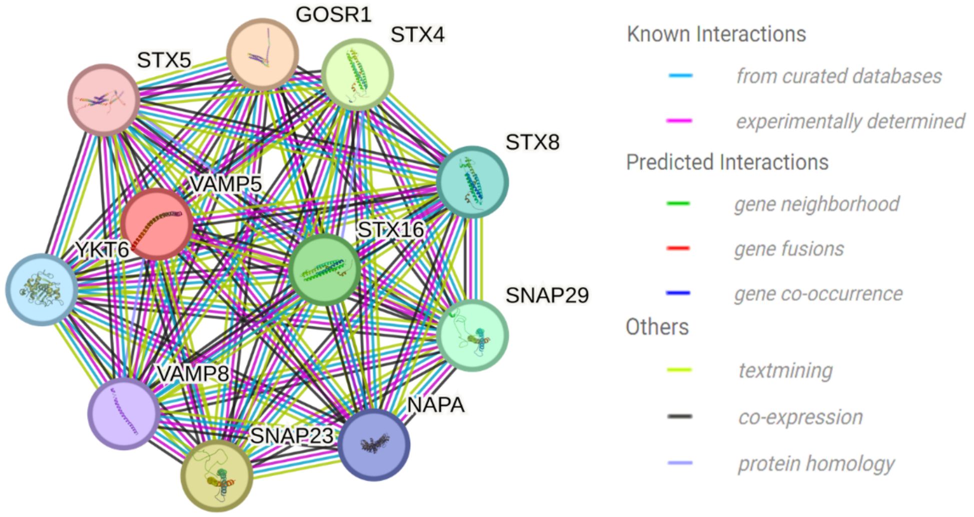

Figure S5. Protein-protein interaction network analysis of VAMP5 with 10 SNARE family genes.
